# Uncovering rearrangements in the Tibetan antelope via population-derived genome refinement and comparative analysis with homologous species

**DOI:** 10.3389/fgene.2024.1302554

**Published:** 2024-02-15

**Authors:** Jiarui Chen, Shuwen Wang, Dong Wang, Yunkang Chiu, Nan Yang, Xinming Lian, Zicheng Zhao, Qing Wei

**Affiliations:** ^1^ College of Eco‐Environmental Engineering, Qinghai University, Xining, Qinghai, China; ^2^ State Key Laboratory of Plateau Ecology and Agriculture, Qinghai University, Xining, Qinghai, China; ^3^ Key Laboratory of Adaptation and Evolution of Plateau Biota, Northwest Institute of Plateau Biology, Chinese Academy of Sciences, Xining, Qinghai, China; ^4^ School of Geographical Science, Qinghai Normal University, Xining, Qinghai, China; ^5^ Shenzhen Byoryn Technology Co., Ltd., Shenzhen, China; ^6^ National Genomics Data Center & CAS Key Laboratory of Genome Sciences and Information, Beijing Institute of Genomics, Chinese Academy of Sciences, Beijing, China; ^7^ Qinghai Provincial Key Laboratory of Animal Ecological Genomics, Xining, Qinghai, China

**Keywords:** *Tibetan antelope*, genome refinement, genome comparison, genome rearrangements, linkage disequilibrium

## Abstract

**Introduction:** The Tibetan antelope (*Pantholops hodgsonii*) is a remarkable mammal thriving in the extreme Qinghai-Tibet Plateau conditions. Despite the availability of its genome sequence, limitations in the scaffold-level assembly have hindered a comprehensive understanding of its genomics. Moreover, comparative analyses with other Bovidae species are lacking, along with insights into genome rearrangements in the Tibetan antelope.

**Methods:** Addressing these gaps, we present a multifaceted approach by refining the Tibetan Antelope genome through linkage disequilibrium analysis with data from 15 newly sequenced samples.

**Results:** The scaffold N50 of the refined reference is 3.2 Mbp, surpassing the previous version by 1.15-fold. Our annotation analysis resulted in 50,750 genes, encompassing 29,324 novel genes not previously study. Comparative analyses reveal 182 unique rearrangements within the scaffolds, contributing to our understanding of evolutionary dynamics and species-specific adaptations. Furthermore, by conducting detailed genomic comparisons and reconstructing rearrangements, we have successfully pioneered the reconstruction of the X-chromosome in the Tibetan antelope.

**Discussion:** This effort enhances our comprehension of the genomic landscape of this species.

## 1 Introduction

*Pantholops hodgsonii*, commonly known as the Tibetan antelope, is a species of large mammal native to the Qinghai-Tibet Plateau. It is well adapted to the extreme environmental conditions of the plateau, such as the high altitude and low oxygen levels, and can withstand temperatures ranging from −40°C to 20°C (Schaller, 2000). Tibetan antelopes are known for their soft and warm wool, called shahtoosh, which is highly valued in the fashion industry (Ruan XD et al., 2005). Studying the Tibetan antelope can also provide insights into the evolution and adaptation of animals to extreme environments, which has important implications for our understanding of evolution and biodiversity in general (Xu et al., 2005; Hassanin et al., 2009; Zhang et al., 2013; Ahmad et al., 2016).

The Tibetan antelope genome has been sequenced, and this provides a valuable resource for further research into the genetics and physiology of the species, as well as comparative genomics with other mammals (Ge et al., 2013; Wang et al., 2015). However, the genome draft is in scaffold level with limited assembly quality. The insufficient resolution of the genome reference introduces obstacles in accurately identifying and characterizing genomic features (Baker, 2012; Mahmoud et al., 2019; Smits, 2019). This hampers our ability to accurately identify structural variants, transposable elements, and epigenetic modifications, thus impacting our understanding of gene regulation and genome function.

Genome comparison is an important tool for understanding the biology, evolution, and diversity of species (Delcher et al., 2002; Emes et al., 2003; Pennacchio, 2003). It can be used to reconstruct evolutionary relationships between mammalian species, providing insights into the diversification and adaptation (Chen et al., 2019). As an important species in bovid species, previous studies have reported partial genome comparison on Sable Antelope using next-generation sequencing (NGS). A study published in 2020 have partial compared Sable Antelope transcriptomic data generated from Sable Antelope skin samples and compared the data to those of other antelope species (Glover et al., 2020; Gooley et al., 2020). The researchers identified several genes that were differentially expressed in Sable Antelope skin and suggested that these genes may be involved in adaptations to the arid and semi-arid environments where Sable Antelope live. As a unique species with adaptability in high attitude in Bovidae family, Tibetan antelope is still lack of genome comparison study in whole genome level.

Genome rearrangements, such as chromosomal inversions, translocations, and duplications, can play a significant role in the evolutionary history of organisms. For example, Zhao studied large-scale rearrangements between human, rat and mouse (Zhao et al., 2004). The study reveals the ancestor components of the three species as well as chromosomal changes through evolution. Li’s research uncovered that significant chromosomal alterations can induce changes in genome-wide expression, consequently fostering adaptation and speciation in Gayal (*Bos frontalis*) (Li et al., 2023); Furthermore, the structural rearrangements observed between taurine cattle and yak offer insights into the high-altitude adaptability of yaks, holding substantial implications for comprehending how large mammals and humans response physiologically and pathologically to hypoxia (Gao et al., 2022). In a separate study, Anthony V. Signore and Jay F. Storz (Signore and Storz, 2020) conducted a comparative genomic analysis of the β-globin gene across ten bovid species, which was found to enhance the blood-O_2_ affinity of Tibetan antelope.

LD is the non-random association of alleles at different loci in population genetics (Good, 2022), thereby can provide distance information in genome assembly. Computational tools have also been developed for genome scaffolding with population resequencing data based on LD (Zhao et al., 2020). In this research, we gathered a total of 15 fresh placental and muscle samples obtained from Tibetan antelope populations in the Kekexili and Arjin regions, which were subsequently subjected to genome resequencing. Utilizing single nucleotide variants identified within the population data, we refined the draft genome of Tibetan Antelope. This refinement resulted in an impressive scaffold N50 of 3.2 Mbp, representing a significant 1.15-fold enhancement over the previous version. Using the MAKER (Cantarel et al., 2008) pipeline for gene annotation on the updated reference genome, we successfully identified a total of 50,750 genes, which includes 29,324 novel genes not reported in the 2013 study (Ge et al., 2013). To evaluate the quality of our annotation, we utilized the BUSCO tool (Seppey et al., 2019) with a mammalian database, resulting in a completeness score that showed an improvement in annotation quality in comparison to the prior version. Furthermore, through genome comparisons, we successfully pinpointed 229 distinctive rearrangements and 176 fusion genes within this improved reference genome. Taking advantage of synteny blocks identified in the closely related Bovidae species, specifically the goat, we reconstructed an evolutionary tree that sheds light on the Tibetan antelope’s genetic relationships. Of noteworthy significance, our use of the MGRA rearrangement tool allowed us to accomplish a pioneering reconstruction of the X-chromosome in the Tibetan antelope. This study represents a significant step forward in our understanding of the genomic landscape of this remarkable species.

## 2 Material and methods

Animal care and research procedures were carried out in accordance with the guiding principles for the care and use of laboratory animals, being approved by the China Zoological Society.

### 2.1 Sample collection

Fresh placental tissues and muscle tissues of accidental death individuals were collected from Kekexili region of Sanjiangyuan National Park in Qinghai Province and Arjinshan Nature Reserve in Xinjiang Uygur autonomous region (n = 2 in Kekexili and n = 13 in Arjin). The tissues were preserved in liquid nitrogen until further analysis.

### 2.2 DNA extraction, library construction, and genome sequencing

0.1 g placental or muscle tissue was used for DNA extraction by BGISP-300 (BGI, Shenzhen, China) with a nucleic acid extraction kit (BGI, Shenzhen, China). After DNA extraction, end-repair was carried out by adding end-repair enzymes under the following cycle conditions: 37°C for 10 min and 65°C for 15 min, followed by adaptor ligation with label-adaptor and ligase at 23°C for 20min. After the end-repair and adaptor ligation, PCR was performed to amplify DNA to the desired concentration under the following cycle conditions: 98°C for 2 min, then 12 cycles at 98°C for 15s, 56°C for 15 s, and 72°C for 30 s, with a final extension at 72°C for 5 min. The DNA amplification products were quantified by Qubit^®^ 2.0 (Life Tech, Invitrogen, Carlsbad, CA, United States) using QubitTM dsDNA HS Assay Kits (Life Tech, Invitrogen, United States), and the concentration ≥2 ng/μL was regarded as qualified standards. The volume was calculated according to the concentration of each sample, and each sample of the same mass was mixed by pooling. The fetal chromosome aneuploidies (T21, T18, and T13) detection kit (Combinatorial Probe-Anchor Synthesis Sequencing Method, CPAS) (BGI, Shenzhen, China) was used for library construction.

Double-strand DNA was thermally denatured into single-strand after pooling, followed by the addition of cyclic buffer and ligase to create DNA circles by cyclization. Qualified DNA circles were used to make DNA Nanoballs (DNBs) by rolling-circle replication. The concentration of DNBs was quantified by Qubit^®^ 2.0 using QubitTM ssDNA assay kits (Life Tech, Invitrogen, United States), and DNBs concentrations within the range of 8–40 ng/μL were considered appropriate. Then, DNBs were loaded onto chips and sequenced on a BGISEQ-500 sequencing platform (BGI, Shenzhen, China) by the SE35 model.

### 2.3 Sequencing alignment and variant calling

We aligned the sequencing reads to the Tibetan antelope genome (GenBank: GCA_000400,835.1) by BWA-MEME2 (https://github.com/bwa-mem2/bwa-mem2) (Jung and Han, 2022). After removing duplicated reads by Picard (version 2.27.5; http://broadinstitute.github.io/picard) (Khan, 2013), Picard (version 1.9) was used to calculate the sequencing coverage along the reference genome. The average sequencing depth of the15 samples in this study was 30.74 folds. We further applied the GATK (version: 4.0) (McKenna et al., 2010) best practice to call somatic variants of the sequenced samples. The resulting alignments are processed with the GATK Base Quality Score Recalibration (BQSR) tool to correct for known biases and sequencing errors. GATK’s Haplotype Caller tool is used to call somatic variants, including single nucleotide variants (SNVs) and small insertions and deletions (indels). Only binary SNPs were remained for further process. The called variants were first filtered by GATK VQSR (Variant Recalibrator and Apply Recalibration). After that we obtained a set of genomic loci deemed reliable for population genetic analysis via several approaches: i) Only bases with an Illumina base quality of at least 20 were included; ii) Only reads with a BWA mapping quality of at least 13 were included; iii) SNPs with a missing data rate less than 15% and a minor allele count greater than three were kept.

### 2.4 Genome refinement

**FIGURE 1:**
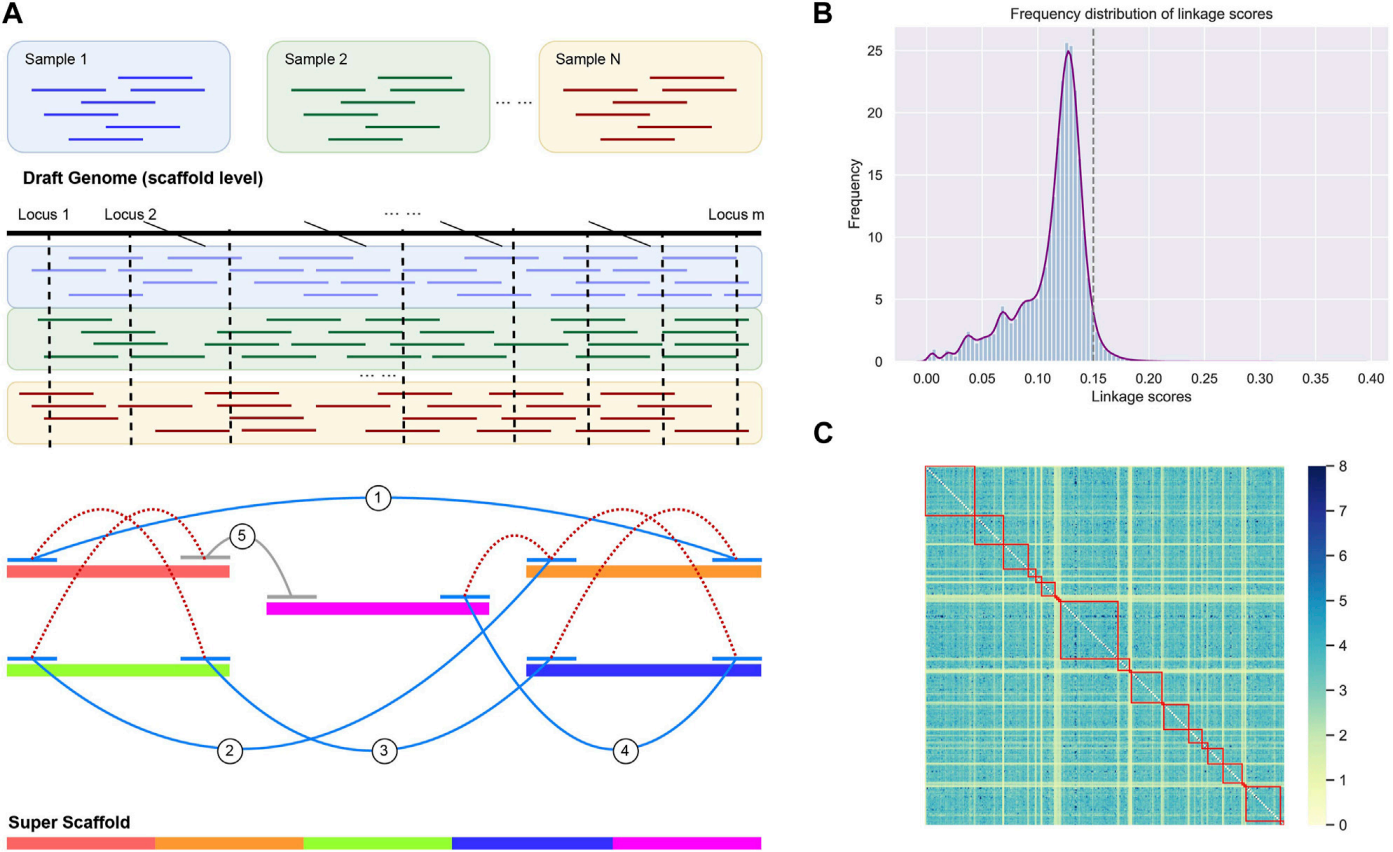
**(A)** The workflow of genome rearrangement. We utilized the Linkage disequilibrium (LD) of the identified single nucleotide variants by resequencing data. **(B)** The distribution of LD scores between scaffold ends. We adopt the score threshold as 0.15, while scores below the threshold were considered as random noise (97% confidential interval). **(C)** The heatmap for the LD score matrix with element intensity in the matrix indicates the strength of LD. The linked scaffolds were highlighted by red squares.

The genome refinement was performed by LDScaff (version 1.0) (Zhao et al., 2020), which is a software for draft genome scaffolding incorporating linkage disequilibrium information in population data. Figure 1A demonstrated the basic workflow of the draft refinement. The linkage scores estimated pairwisely between scaffold ends. LDScaff determines the order and orientation of the original scaffolds by a graphical algorithm. It takes a constant number of SNPs from two edges of the original scaffolds as input. An in-house scripted was used to transform the variant file in *vcf* format to LDScaff input. The number of SNPs at scaffold edge was set to 500. We implemented LDScaff to construct the scaffolding graph, calculate the pairwise linkage score of the original scaffold edges, and determine the permutation and orientation of the original scaffolds. The original scaffolds were linked to one or several circles by the constructed scaffold graph. The circles were transformed into linear scaffolds by removing edges with low linkage score. The threshold was determined by the overall distribution of the linkage scores calculated in the scaffold graph. To evaluate the quality of assemblies, we used QUAST (Gurevich et al., 2013) to collect various metrics (command line: “–eukaryote–min-contig 3,000 –min-alignment 500 –extensive-mis-size 7,000 –fast–split-scaffolds”).

### 2.5 Genome annotation

During the refinement of the Tibetan antelope reference genome, we also conducted gene annotation employing the Maker3 (version: 3.01.04) pipeline. Inputs critical for the annotation included: 1) a repeat model derived from RepeatModeler (version: 2.0.2) with the refined reference as input; 2) Protein homology evidence obtained from protein sequences of closely related Bovidae species, including *Bos mutus* (Accession number: GCA000298355.1), *Capra hircus* (Accession number: GCA026652205.1), and *Ovis aries* (Accession number: GCA000298735.2); 3) Expressed sequence tags (ESTs) evidence from prior RNA studies of the Tibetan antelope (Accession number: GCA_000400835.1).

The initial stage of annotation utilized Maker, incorporating RepeatMasker for repeat elements, and integrating EST and protein homology evidence to annotate genes. Subsequently, we re-annotated using a SNAP-trained GFF file from Maker’s former round outputs, proceeding with two additional rounds to refine the annotations. This iterative approach led to a polished dataset, which was then subjected to augmented *ab initio* gene prediction using AUGUSTUS, driven by the closest available species profile, which in this case was human—due to the absence of Tibetan antelope-specific protein or RNA-sequence data. To assess the quality of our annotation, we utilized BUSCO with a mammalian database.

### 2.6 Genome comparison analysis

We obtained the genome sequences of eight organisms, specifically *O. aries*, *Capra hirus*, *Capra sibirica*, *Pseudois nayaur*, *Hippotragus equinus*, *Hippotragus niger*, *Procapra przewalskii* and *B. mutus* from NCBI with reference numbers detailed in Table 1. To facilitate genome comparison, we employed Mummer (version: 4.0) (Darling et al., 2018) with default parameters for alignment between these reference genomes and the Tibetan Antelope reference. The alignment process comprised by the following two steps: 1. the genomes are indexed using the *nucmer* command; 2. the actual alignment is performed using the *show-coords* command.

**TABLE 1:** The list of organisms analyzed in this study and their draft genome assembly information.

Organism	Assembly accession	Assembly level	Submitter	Date	Chromosome number (if applicable)
*Ovis aries*	RefSeq: GCF_016772045.1	Chromosome	University of Idaho	2021/2/3	27
	Genbank: GCA_016772045.1				
*Capra hircus*	RefSeq: GCF_001704415.2	Chromosome	USDA ARS	2016/8/24	30
	Genbank: GCA_001704415.2				
*Hippotragus equinus*	Genbank: GCA_016433095.1	Scaffold	CIBIO, University of Porto	2020/12/29	N.A.
*Hippotragus niger*	Genbank: GCA_006942125.1	Scaffold	Saint-Petersburg State University	2019/7/12	N.A.
*Bos mutus*	RefSeq: GCF_000298355.1	Scaffold	BGI-shenzhen	2013/1/9	N.A.
	Genbank: GCA_000298355.1				
*Pseudois nayaur*	Genbank: GCA_003182575.1	Scaffold	Northwest A&F University	2018/5/31	N.A.
*Capra sibirica*	Genbank: GCA_003182615.2	Scaffold	Northwest A&F University	2018/10/30	N.A.
*Procapra przewalskii*	Genbank: GCA_006410515.1	Scaffold	Northwestern Polytechnical University	2019/6/24	N.A.
*Pantholops hodgsonii*	Genbank: GCA_000400835.1	Scaffold	BGI	2013/5/28	N.A.

The results generated by Mummer comprise a comprehensive list of aligned regions, encompassing their coordinates and similarity scores. However, this output may contain smaller alignments that lack biological significance and do not represent genuine homologous relationships between the genomes. To enhance the quality of our analysis, we implemented two filters: a size filter and a similarity filter. Specifically, we excluded blocks measuring less than 2000 base pairs and those exhibiting a similarity score below 90%. For visualization purposes, we employed dot plots and circus plots. Dot plots were generated using the dotPlotly (https://github.com/tpoorten/dotPlotly) R script “mummerCoordsDotPlotly,” while circus plots were created using an in-house R script.

### 2.7 Genome syntonies and phylogeny tree inference

For individual pairwise comparison, a rigorous filtration process was conducted. The objective was to discern genome syntonies shared by Sable Antelope and all eight other organisms. An in-house script facilitated this task. Notably, regions demonstrating an overlap exceeding 80% were categorized as identical regions. These identified blocks were consequently regarded as homologous sequences shared across the species. Subsequently, the homologous sequences were isolated and subjected to a base-to-base multiple sequence alignment, executed using MAFFT (version 7) (Katoh and Standley, 2013) with default parameters and the auto mode. The resulting alignment data served as input for the construction of a phylogenetic tree using BEAST4 (Drummond and Rambaut, 2007). The BEAST4 software was configured with default settings, employing the Yule Process as the tree prior.

### 2.8 Genome rearrangement and fusion gene analysis

As *O. aries* and *Capra Hirus* have chromosome level assembly, we performed genome rearrangement analysis between Tibetan Antelope, *O. aries* and *Capra Hirus* by Multiple Genome Rearrangements and Ancestors (MGRA). MGRA is a software tool for inferring the evolutionary history of a set of genomes by analyzing genome rearrangements. By considering the syntony blocks in Tibetan antelope as serial numbers, we order the blocks by indexes in *O. aries* and *Capra Hirus*. MGRA was executed with default parameters, yielding several output files. Among these, a tree file was generated to elucidate the inferred evolutionary history of the genomes. Additionally, a list detailing the rearrangement operations inferred to have occurred along each branch of the tree was also produced.

We also performed gene fusion analysis with the GriffinDetector program, employing a phylotree that encompassed Tibetan antelope, *B. mutus*, *Capra hircus*, and *O. aries*, all of which had corresponding annotated protein sequences and GFF3 files. The program aligned RNA-seq data to the reference genome and, by integrating the phylotree and GFF3 annotation files, accurately identified fusion candidates based on changes in gene structure and expression patterns. We performed GriffinDetector with default parameters, with *B. mutus* treated as outgroup species.

## 3 Results

### 3.1 Genome refinement by linkage disequilibrium and genome annotation

In this study, we apply the LDScaff method to refine the draft reference genome of the Tibetan antelope (*P. hodgsonii*). Through whole-genome sequencing of the fifteen Tibetan antelope samples, we meticulously identify genetic variants and employ linkage disequilibrium (LD) analysis from both ends of the draft scaffolds. By leveraging LD information, we effectively link the scaffolds, leading to an improved and more accurate genome reference for this species.

The scaffold filtering process was undertaken before executing the LDScaff algorithm, wherein two conditions were applied: 1. Scaffolds with a length shorter than 20,000 bp were excluded, and 2. Scaffolds with fewer than 100 identified Single Nucleotide Polymorphisms (SNPs) were also filtered out. Following the filtering process, 166 scaffolds remained, which were subsequently linked into sets of cycles using varying cutoff thresholds, as illustrated in Table 2. The pairwise linkage scores were computed while constructing the complete graph based on scaffold terminals. The distribution of these linkage scores was depicted in Figure 1B, revealing a Gaussian distribution for the random linkage scores, centered around a mean value of approximately 0.12, with a 97% confidence interval ranging from 0.09 to 0.15.

**TABLE 2:** Scaffold refinement of Tibetan Antelope with LDScaff by variant LD score cutoff.

LD cutoff	Scaffold no. before	Scaffold no. after	N50 before	N50 after
0.1	3,037	2,852	2.772,860	3,288,346
0.15	3,037	2,867	2,772,860	3,288,346
0.2	3,037	2,872	2,772,860	3,283,285
0.25	3,037	2,876	2,772,860	3,269,951
0.3	3,037	2,952	2,772,860	3,069,389
0.35	3,037	3,011	2,772,860	2,848,742
0.4	3,037	3,030	2,772,860	2,814,880

LDScaff utilized the linkage scores, as depicted in Figure 1A, to determine the permutation and orientation of the scaffolds, thus forming cycles. A critical parameter for LDScaff is the cutoff threshold of the linkage scores, as it serves to eliminate the weakest links and form super scaffolds. Based on the observations from Figure 1B, we determined the super scaffold cutoff threshold to be 0.15. The heatmap analysis, as presented in Figure 1C, provided valuable insights into the linkage scores within the super scaffolds. Higher scores in the heatmap denoted the reliability of the linked scaffolds, and it was evident from the heatmap that the diagnostic lines primarily exhibited elevated values, thus indicating that the linked scaffolds achieved higher linkage scores through our methodology.

The reference refinement process yielded significant improvements in the N50 metric, resulting in a value of 3.6 MB, which was approximately 2.5 times larger than the original assembly (1.3 MB). The qualities of both the PHIL assembly and the refined assembly were assessed using QUAST, with the relevant metrics detailed in Table 3.

**TABLE 3:** The draft reference statistics accessed by QUAST before and after reference refinement.

Assembly	GCA_000400,835.1_PHO1.0_genomic	Refined reference
# contigs (≥0 bp)	15,058	14,944
# contigs (≥1,000 bp)	5,395	5,281
# contigs (≥5,000 bp)	3,037	2,923
# contigs (≥10,000 bp)	2,558	2,444
# contigs (≥25,000 bp)	2,118	2004
# contigs (≥50,000 bp)	1883	1769
Total length (≥0 bp)	2,696,869,832	2,795,093,702
Total length (≥1,000 bp)	2,691,424,541	2,789,648,411
Total length (≥5,000 bp)	2,686,953,467	2,785,177,337
Total length (≥10,000 bp)	2,683,532,326	2,781,756,196
Total length (≥25,000 bp)	2,676,487,241	2,774,711,111
Total length (≥50,000 bp)	2,668,305,838	2,766,529,708
# contigs	3,434	3,320
Largest contig	13,453,139	56,218,888
Total length	2,688,433,699	2,786,657,569
N50	2,774,095	3,198,051
N90	715,120	761,415
#N’s per 100 kbp	6958.02	6940.83

The reference genome of the Tibetan Antelope has undergone a reannotation process to update the delineation of its repeat elements and genes. We annotated 50,750 genes for the refined reference. Among the annotated genes, 29,324 genes had not been previously identified within the genomic data for this species. To assess the quality and completeness of the updated assembly and the accompanying annotation, we utilized the Benchmarking universal Single-Copy Orthologs (BUSCO) analysis with a mammalian-specific database. Results of this assessment are detailed in Table 4. Our evaluation indicated a quantifiable improvement in both the assembly and annotation of the Tibetan Antelope’s genome. The metrics highlighted an increase in completeness, signifying an advancement over prior versions in terms of genomic resolution and the depth of genetic information represented in the reference.

**TABLE 4:** BUSCOs for the refined reference assembly and annotation compared to previous study.

Genome version		GCA_000400,835.1_PHO1.0	Refined reference
Complete BUSCOs (C)	Assembly	8,328	8,332
Fragmented BUSCOs (F)		413	413
Missing BUSCOs (M)		485	481
Total BUSCO groups searched (n)		9,226	
Complete BUSCOs (C)	Proteins	8,641	8,698
Fragmented BUSCOs (F)		334	324
Missing BUSCOs (M)		251	204
Total BUSCO groups searched (n)		9,226	

### 3.2 Evolutionary history of tibetan antelope in bovidae species

**FIGURE 2:**
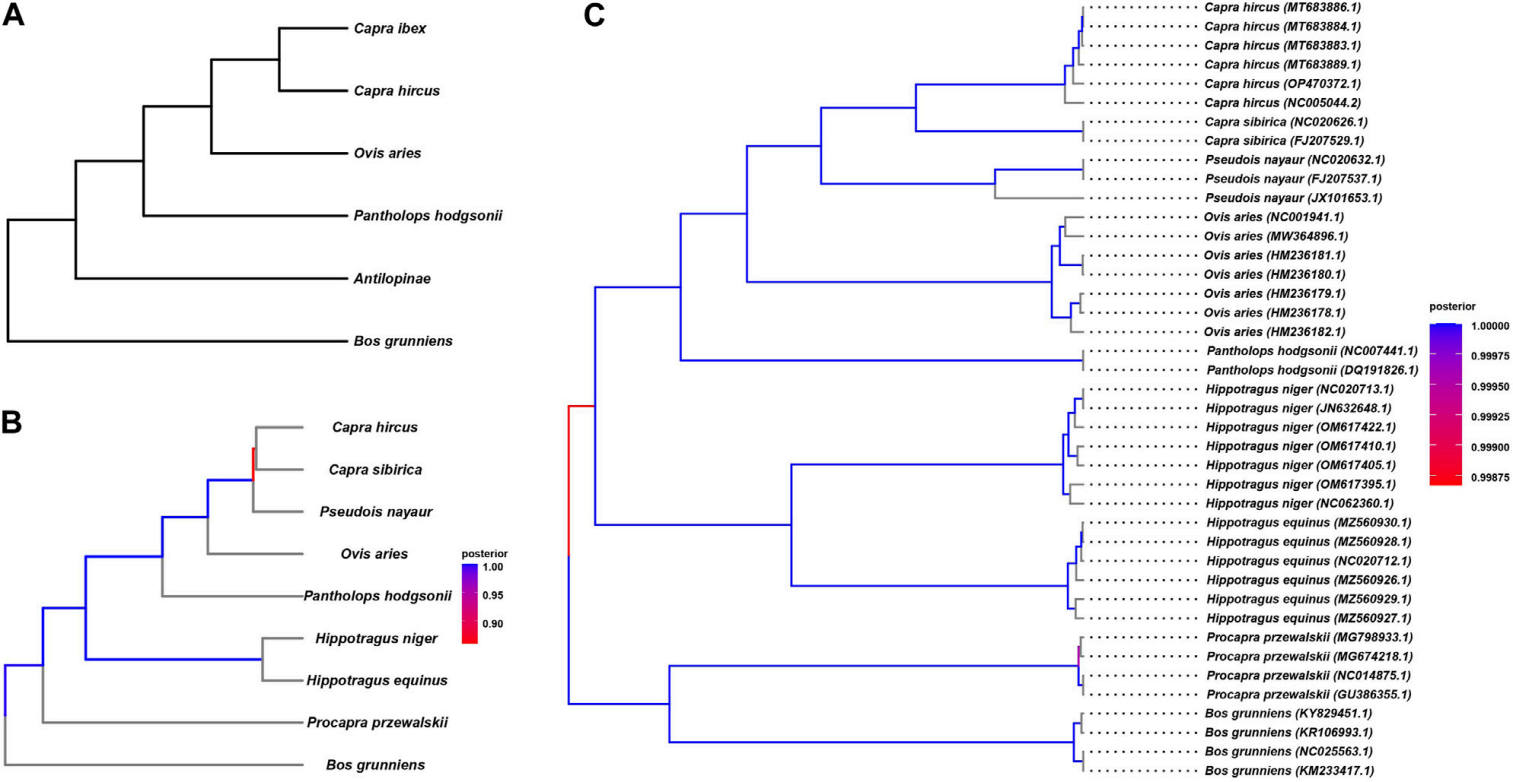
The polygenetic tree constructed by variant methods. **(A)** Species tree reported by previous study, constructed by genome wide comparison. **(B)** The polygenetic tree constructed based homologous regions. **(C)** The polygenetic tree constructed based on mtDNA.

We endeavored to elucidate the evolutionary history of the Tibetan antelope at the whole-genome level by constructing a phylogenetic tree encompassing nine organisms, including the Tibetan antelope, as detailed in Table 1. To achieve this, we employed various variant methods, as illustrated in Figure 2. In the previous large-scale genetic comparison of Bovidae species, we specifically extracted the species utilized in the study (Figure 2A). The resulting topology of the phylogenetic tree was considered as the gold standard for comparison purposes.

We constructed the phylogenetic tree by considering homogenetic regions across the species (Figure 2B). Our approach involved conducting pairwise genome alignments, through which we identified a total of 35,874 blocks with an average length of 8,465 commonly present in all nine species. These regions, denoted as homologous regions, were meticulously extracted, and utilized to construct the phylogenetic tree employing the BEAST 4 software. The detailed outcomes of the pairwise alignments are presented in Table 5. By leveraging these homologous regions and incorporating the BEAST 4 analysis, we aimed to unravel the evolutionary relationships and divergence patterns among the Tibetan antelope and the other studied organisms.

**TABLE 5:** Statistics for pairwise genome wide alignment for variant organisms *versus* Tibetan Antelope.

Organism	In *Pantholops hodgsonii* (Tibetan antelope) (%)	In comparison organism (%)	Block no.	Average length of blocks
*Ovis aries* (*Sheep*)	15.08	15.57	35,874	11,350
*Capra hircus* (*Goat*)	15.72	15.1	36,367	11,661
*Hippotragus equinus* (*Roan antelope*)	11.17	11.58	27,860	10,817
*Bos mutus*\ (*Wild yak*)	0.31	0.32	1,039	8,071
*Pseudois nayaur* (*Bharal*)	14.1	14.72	37,178	10,231
*Capra sibirica* (*Siberian ibex*)	14.74	14.55	32,044	12,411
*Procapra przewalskii* (*Gazelle*)	8.35	8.35	26,572	8,465
*Hippotragus niger* (*Sable antelope*)	7.08	7.36	17,163	11,134

The constructed tree shows consistent topology structure compared to Figure 2A. We can observe that *P. przewalskii* and *Bos grunniens*, both yak-related, form distinct branches, indicating their shared ancestry. On a separate branch, the Tibetan antelope clusters with *O. aries*, *P. nayaur*, *Capra sibirica*, and *Capra hircus*—all of which belong to the *Ovis* genus. The clustering of the Tibetan antelope alongside *O. aries* within the same clade underscores their close evolutionary ties and potential shared history. In contrast, *Capra hircus* branches off into a separate subclade, indicating a relatively more distant genetic association with the Tibetan antelope. These two species stand out as the most recent common ancestors when compared to the Tibetan antelope.

mtDNA is predominantly inherited through the maternal lineage, and it often exhibits differences when compared to the nuclear DNA (commonly referred to as autosomal DNA) in phylogenetic tree construction, sometimes showing significant disparities. These distinctions can be explained by the genetic processes involving sex selection, genetic drift during migration, and the dominant sex in the population (such as in patrilineal or matrilineal societies). For instance, previous studies on the migration of donkeys and horses revealed substantial discrepancies between autosomal and mtDNA-based phylogenetic trees, possibly due to tribes with dominant male individuals possessing higher levels of mating power during migration and population admixture. Therefore, we also performed mtDNA-based phylogenetic analysis. We obtained mtDNA sequences for all nine species from public databases, and the data list can be found in Supplementary Table S1. Utilizing BEAST4, we constructed the evolutionary tree (Figure 2C) based on mtDNA. The results of mtDNA-based tree construction were generally consistent with the autosomal-based results, indicating that the evolutionary history of the Tibetan antelope likely involved fewer instances of population admixture and migration when compared to domesticated animals like donkeys and horses. This concurs with the adaptation of the Tibetan antelope to harsh high-altitude environments.

### 3.3 Genome comparison reveals structure rearrangements in tibetan antelope

**FIGURE 3:**
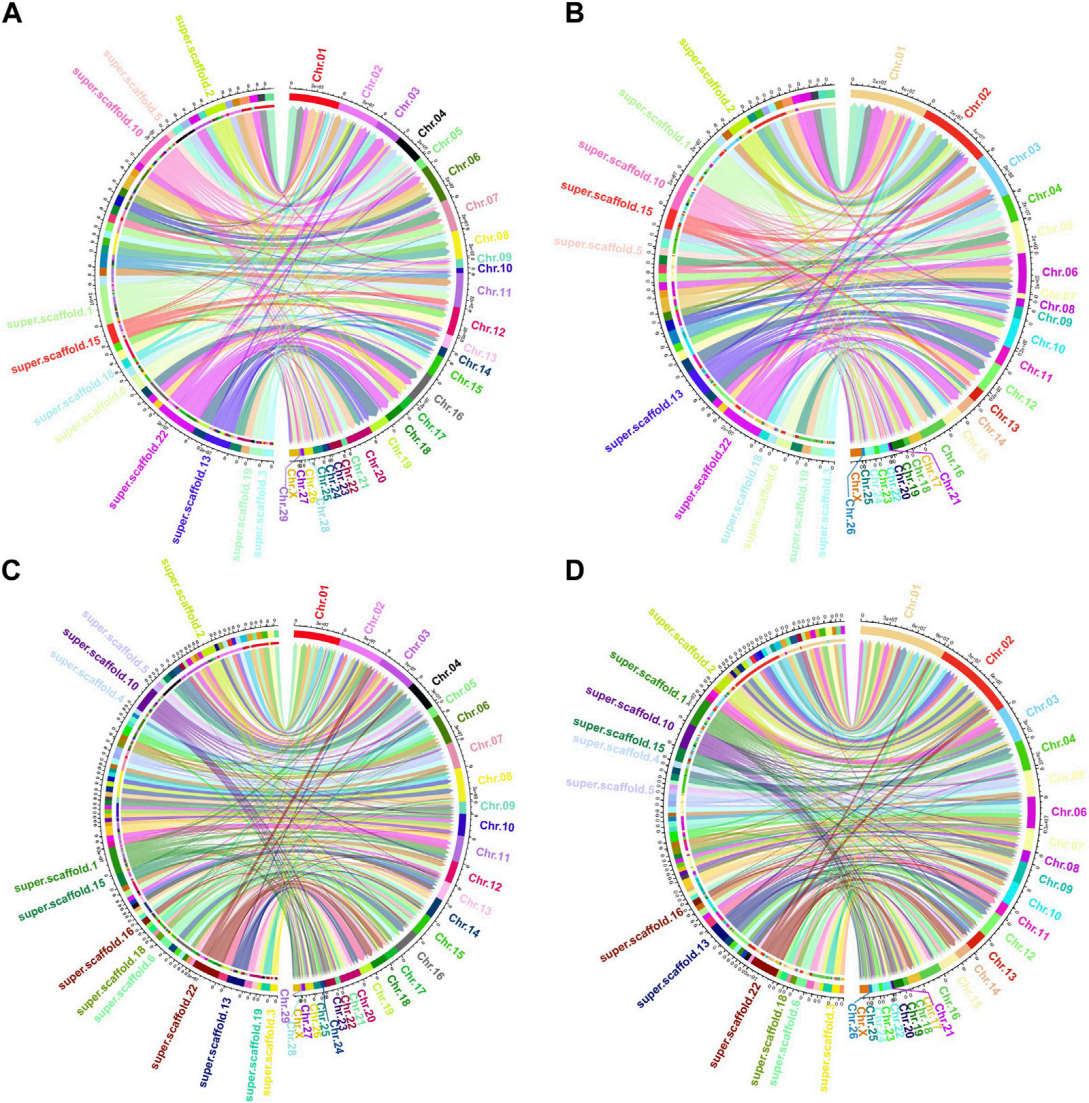
Circos plot of the aligned homologous regions for refined *Pantholops hodgsonii* reference versus *Capra hircus* and *Ovis aries* with top 50 and top 100 longest scaffolds. **(A)** top 50 longest scaffolds versus *Capra hircus*; **(B)** top 50 longest scaffolds versus *Ovis aries*; **(C)** top 100 longest scaffolds versus *Capra hircus*; **(D)** top 100 longest scaffolds versus *Ovis aries*.

Among the genomes compared, *O. aries* and *Capra hircus* boast chromosome-level reference drafts. Consequently, we proceeded to visualize the homologous regions of the Tibetan antelope when compared to these two species, as depicted in Figure 3. To enhance clarity, we organized the antelope scaffolds by length and visualized them separately, focusing on the top 50 and top 100 scaffolds.

The visualization of these homologous regions revealed intriguing chromosome-level rearrangements, notably inversions and translocations. Notably, the sequence of Tibetan antelope scaffolds aligned to the same chromosomes as those of the two homologous species appeared in different orders. This observation suggests that rearrangements are a common occurrence in the evolutionary history of the Tibetan antelope.

**FIGURE 4:**
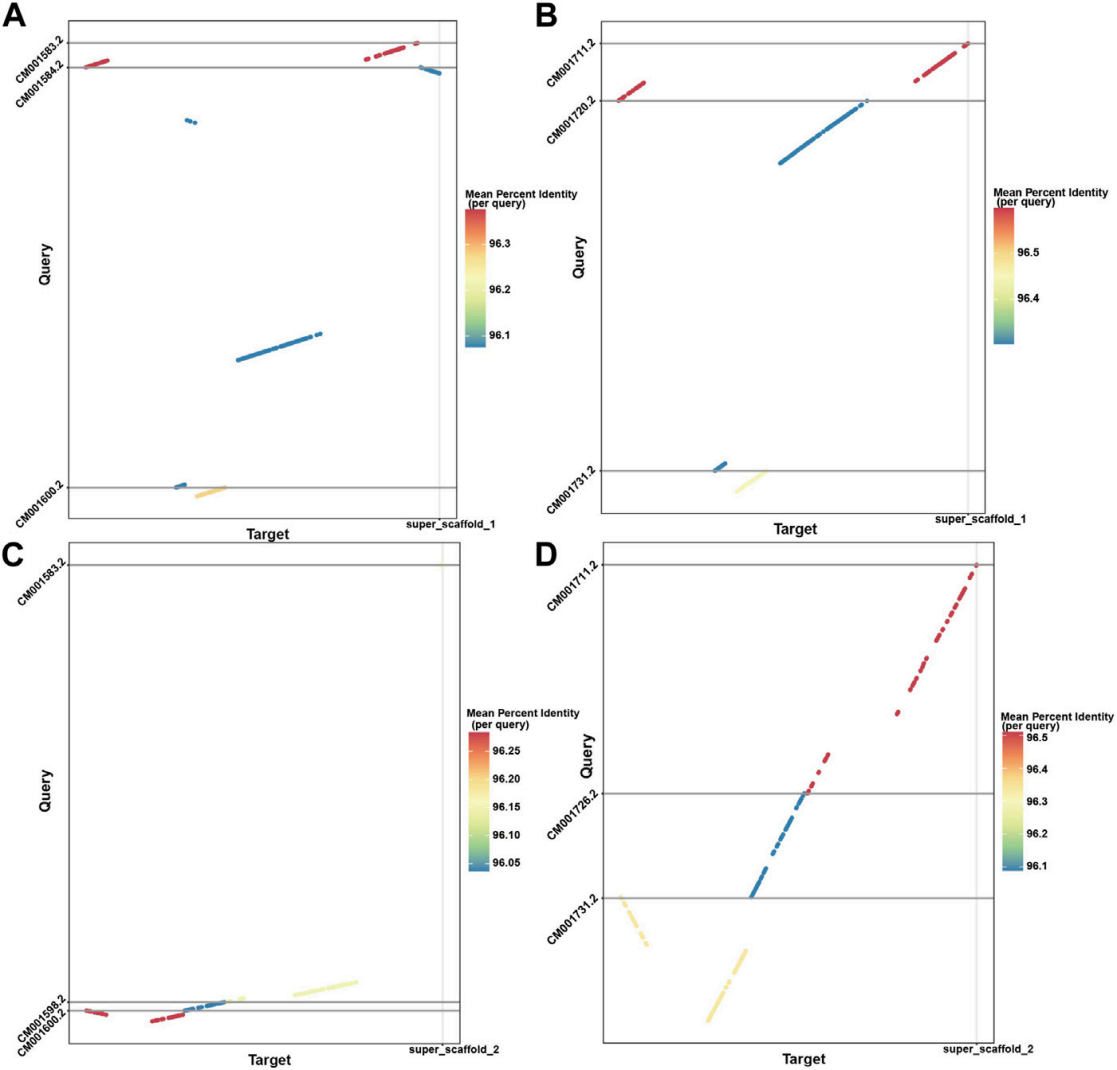
The dot plots demonstrated the inversion and translocations in Tibetan Antelope compared to *Capra hircus* and *Ovis aries*; **(A)** super scaffold 1 versus *Capra hircus*; **(B)** super scaffold 1 versus *Ovis aries*; **(C)** super scaffold 2 versus *Capra hircus*; **(D)** super scaffold 2 versus *Ovis aries*.

With the powerful capability of identifying rearrangements in LDScaff scaffolding, we conducted a visual comparison of the super scaffolds assembled by LDScaff with respect to the two chromosome-level homologous species, *O. aries* and *Capra hircus*, as depicted in Figure 4. Figures 4A, B illustrate the dot plot of super scaffold 1 against *O. aries* and *Capra hircus*, respectively, while Figures 4C, D depict the dot plot of super scaffold 2 against the same homologous species. In the case of super scaffold 1, its alignment to specific regions in *O. aries* and *Capra hircus* revealed the occurrence of inversions in the Tibetan antelope’s genome. Specifically, the last syntony block in super scaffold 1 demonstrated a reversal, indicating a significant inversion event in the evolutionary history of the Tibetan antelope. Through genome refinement and revision, we identified 229 rearrangements in the refined scaffolds of Tibetan antelope, including 135 inversions and 94 translocations. The detail statistics of rearrangements identified were listed in Supplementary Table S2.

Fusion gene events in the Tibetan antelope were characterized relative to two comparator species, employing *B. mutus* as an outgroup and utilizing GriffinDetector for the detection process. This analysis yielded a count of 21 fusion genes when contrasted with *Capra hircus*, and an additional 71 fusion genes in comparison with *O. aries*. Within this dataset, 176 genes were flagged as potential fusion events with both comparator species. A comprehensive inventory of these candidate fusion genes is provided in Supplementary Table S3.

As the Tibetan Antelope lacks a chromosome-level assembly, we utilized mummer to identify synteny blocks from the homologous regions shared among the three species, namely, *O. aries*, *Capra hircus*, and the Tibetan Antelope. We applied the following criteria for identification: 1) The regions in all three species should exhibit more than 80% overlap, and 2) The synteny block should be longer than 10,000 base pairs. As a result, 1,331 identity blocks were identified through our methodology.

**FIGURE 5:**
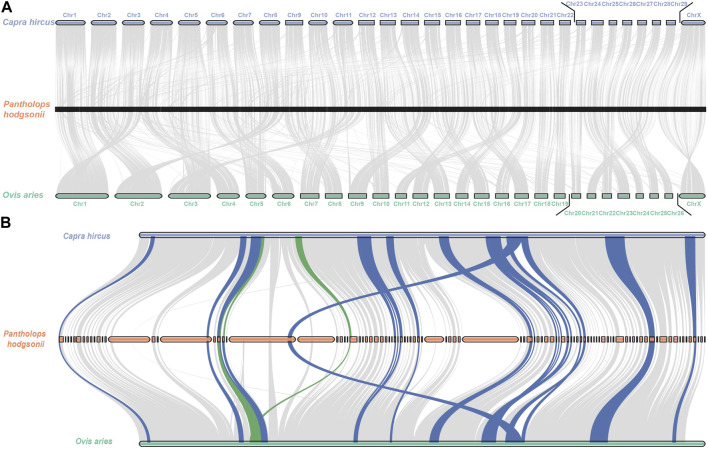
Rearrangement reconstruction of the consensus blocks in *Pantholops hodgsonii* **(A)** in whole genome level; **(B)** in chromosome X.

Based on the evolutionary history topology inferred from the three species, we subsequently reconstructed the permutation and synteny blocks in the Tibetan Antelope using the Multiple Genome Rearrangement Algorithm (MGRA), as depicted in Figure 5A. These regions represent candidate rearrangements in the Tibetan Antelope’s genome. We also reconstructed the syntony blocks in chromosome X. According to the previous defined block filtration, we identified 135 syntony blocks in chromosome X. The reordered syntony blocks were shown in Figure 5B. The adopted scaffolds and their corresponding order are listed in Supplementary Table S4. The identification of these candidate rearrangements provides a foundation for further investigations into the genetic mechanisms underlying the adaptation and diversification of this fascinating species.

## 4 Discussion and conclusion

The Tibetan antelope is well-known for its ability to thrive in high altitudes with low oxygen levels and extreme temperature fluctuations. Tibetan antelope reference genome provides a resource for research in genetics, physiology, and comparative genomics. However, the genome draft being at the limited level poses challenges in accurately identifying and characterizing genomic features, including structural variants, transposable elements, and epigenetic modifications.

In this study, we employed the concept of linkage disequilibrium (LD) in population genetics. LD provides valuable distance information for genome assembly and enables genome scaffolding using population resequencing data. Leveraging this approach, we successfully refined the draft genome of the Tibetan antelope and compared it with other Bovidae species, some of which have chromosome level genomes. This comparison provided valuable insights into genomic similarities and differences among these species. Additionally, we conducted genome rearrangement analysis using the MGRA algorithm to explore potential rearrangements in the evolutionary history of the Tibetan antelope compared to other species.

The challenges associated with sample collection from the Tibetan antelope, including their remote habitat, restricted geographical range, and conservation considerations, posed constraints on the feasibility of employing hybrid assembly methods such as long reads and Hi-C sequencing. Legal and ethical restrictions further limited our ability to pursue these approaches. Faced with these practical constraints, we opted for the LD-based scaffolding method, leveraging population-level genetic information. This choice not only provided a cost-effective alternative but also offered an efficient means to enhance the draft genome without the need for extensive sample collection or substantial financial investment. While we acknowledge the advancements in next-generation sequencing (NGS) technologies, we firmly believe that, given the specific challenges of our study, the LD-based approach represents a justified and pragmatic choice, striking a balance between reliability and practical feasibility.

Genome rearrangements are crucial events shaping the genetic landscape, with significant implications in genomics and evolutionary biology. They serve as molecular markers for reconstructing evolutionary history and understanding divergence patterns, contributing to the remarkable diversity of life. Rearrangements play a key role in species adaptation and speciation, generating genetic variations that drive adaptive potential and survival in changing environments (Butlin, 2005; Lexer and Widmer, 2008). They influence population structure and dynamics, shedding light on genetic architecture, historical demography, gene flow, and differentiation.

The foundation of organism studies, including the Tibetan antelope, lies in the chromosome-level genome. Previous cytogenetic studies have emphasized the importance of a comprehensive and well-assembled high-quality reference for conducting detailed rearrangement analysis (Ge et al., 2013). While the current genome reference has been refined and candidate rearrangements have been identified, it still has limitations in fully identifying rearrangements. A more refined and accurate genome reference is essential to gain a deeper understanding of the species' evolution, migration patterns, and adaptations to high altitudes. This reference is crucial for exploring the genetic basis of high-altitude adaptability and shedding light on the evolutionary history and unique biology of the Tibetan antelope (Xu et al., 2005). Furthermore, a high-quality genome reference would enable researchers to uncover subtle or rare rearrangements that may play pivotal roles in shaping the species' adaptation to its challenging environment. Therefore, a well-assembled genome reference remains a crucial resource for advancing our knowledge of the Tibetan antelope and its exceptional ability to thrive in extreme conditions.

## Data Availability

The data presented in the study are deposited in the National Genomics Data Center repository, accession number PRJCA021064.

## References

[B1] Ahmad K. Kumar V. P. Joshi B. D. Raza M. Nigam P. Khan A. A. (2016). Genetic diversity of the Tibetan antelope (*Pantholops hodgsonii*) population of Ladakh, India, its relationship with other populations and conservation implications. BMC Res. Notes 9 (1), 477–510. 10.1186/s13104-016-2271-4 27769305 PMC5073904

[B22] Baker M. (2012). De novo genome assembly: what every biologist should know. Nature Methods 9 (4), 333–337. 10.1038/nmeth.1935

[B2] Butlin R. K. (2005). Recombination and speciation. Mol. Ecol. 14 (9), 2621–2635. 10.1111/j.1365-294X.2005.02617.x 16029465

[B3] Cantarel B. L. Korf I. Robb S. M. Parra G. Ross E. Moore B. (2008). MAKER: an easy-to-use annotation pipeline designed for emerging model organism genomes. Genome Res. 18 (1), 188–196. 10.1101/gr.6743907 18025269 PMC2134774

[B26] Chen L. Qiu Q. Jiang Y. Wang K. Lin Z. S. Li Z. P. (2019). Large-scale ruminant genome sequencing provides insights into their evolution and distinct traits. Science 364 (6446). 10.1126/science.aav6202 31221828

[B4] Darling A. E. Marçais G. Delcher A. L. Phillippy A. M. Coston R. Salzberg S. L. (2018). MUMmer4: a fast and versatile genome alignment system. PLOS Comput. Biol. 14 (1), e1005944. 10.1371/journal.pcbi.1005944 29373581 PMC5802927

[B27] Delcher A. L. Phillippy A. Carlton J. Salzberg S. L. (2002). Fast algorithms for large-scale genome alignment and comparison. Nucleic Acids Res. 30, 2478–2483. 10.1093/nar/30.11.2478 12034836 PMC117189

[B5] Drummond A. J. Rambaut A. (2007). BEAST: bayesian evolutionary analysis by sampling trees. BMC Evol. Biol. 7 (1), 214. 10.1186/1471-2148-7-214 17996036 PMC2247476

[B28] Emes R. D. Goodstadt L. Winter E. E. Ponting C. P. (2003). Comparison of the genomes of human and mouse lays the foundation of genome zoology. Hum. Mol. Genet. 12 (7), 701–709. 10.1093/hmg/ddg078 12651866

[B6] Gao X. Wang S. Wang Y. F. Li S. Wu S. X. Yan R. G. (2022). Long read genome assemblies complemented by single cell RNA-sequencing reveal genetic and cellular mechanisms underlying the adaptive evolution of yak. Nat. Commun. 13 (1), 4887. 10.1038/s41467-022-32164-9 36068211 PMC9448747

[B7] Ge R. L. Cai Q. L. Shen Y. Y. San A. Ma L. Zhang Y. (2013). Draft genome sequence of the Tibetan antelope. Nat. Commun. 4 (1), 1858. 10.1038/ncomms2860 23673643 PMC3674232

[B32] Glover B. Macfarlane M. Bengis R. O’Dell J. Steyl J. Heerden H. V. (2020). Investigation of Brucella melitensis in Sable Antelope (Hippotragus niger) in South Africa. Microorganisms 8 (10). 10.3390/microorganisms8101494 PMC7600299 33003292

[B8] Good B. H. (2022). Linkage disequilibrium between rare mutations. GENETICS 220 (4), iyac004. 10.1093/genetics/iyac004 35100407 PMC8982034

[B31] Gooley R. M. Tamazian G. Castañeda‐Rico S. Murphy K. R. Dobrynin P. Ferrie G. M. (2020). Comparison of genomic diversity and structure of sable antelope (Hippotragus niger) in zoos, conservation centers, and private ranches in North America. Evol. Appl. 13 (8), 2143–2154. 10.1111/eva.12976 32908610 PMC7463370

[B9] Gurevich A. Saveliev V. Vyahhi N. Tesler G. (2013). QUAST: quality assessment tool for genome assemblies. Bioinformatics 29 (8), 1072–1075. 10.1093/bioinformatics/btt086 23422339 PMC3624806

[B10] Hassanin A. Ropiquet A. Couloux A. Cruaud C. (2009). Evolution of the mitochondrial genome in mammals living at high altitude: new insights from a study of the tribe caprini (Bovidae, *antilopinae*). J. Mol. Evol. 68 (4), 293–310. 10.1007/s00239-009-9208-7 19294454

[B33] Jung Y. Han D. (2022). BWA-MEME: BWA-MEM emulated with a machine learning approach. Bioinformatics 38 (9), 2404–2413. 10.1093/bioinformatics/btac137 35253835

[B34] Khan S. H. (2013). A Picard-Mann hybrid iterative process. Fixed Point Theory Appl. 69 (2013). 10.1186/1687-1812-2013-69

[B11] Katoh K. Standley D. M. (2013). MAFFT multiple sequence alignment software version 7: improvements in performance and usability. Mol. Biol. Evol. 30 (4), 772–780. 10.1093/molbev/mst010 23329690 PMC3603318

[B12] Lexer C. Widmer A. (2008). Review. The genic view of plant speciation: recent progress and emerging questions. Philosophical Trans. R. Soc. B Biol. Sci. 363 (1506), 3023–3036. 10.1098/rstb.2008.0078 PMC2607315 18579476

[B13] Li Y. Wang S. Zhang Z. Luo J. Lin G. L. Deng W.-D. (2023). Large-scale chromosomal changes lead to genome-level expression alterations, environmental adaptation, and speciation in the gayal (*Bos frontalis*). Mol. Biol. Evol. 40 (1), msad006. 10.1093/molbev/msad006 36625089 PMC9874039

[B23] Mahmoud M. Gobet N. Cruz-Dávalos D. I. Mounier N. Dessimoz C. Sedlazeck F. J. (2019). Structural variant calling: the long and the short of it. Genome Biol. 20 (1). 10.1186/s13059-019-1828-7 PMC6868818 31747936

[B24] McKenna A. Hanna M. Banks E. Sivachenko A. Cibulskis K. Kernytsky A. (2010). The Genome Analysis Toolkit: a MapReduce framework for analyzing next-generation DNA sequencing data. Genome Res. 20 (9), 1297–1303. 10.1101/gr.107524.110 20644199 PMC2928508

[B25] Pennacchio L. A. (2003). Insights from human/mouse genome comparisons. Mamm. Genome 14 (7), 429–436. 10.1007/s00335-002-4001-1 12925891

[B14] Ruan Xd H. P. J. Zhang J. L. Wan Q. H. Fang S. G. (2005). Evolutionary history and current population relationships of the chiru (*pantholops hodgsonii*) inferred from mtDNA. Var. J. Mammal. 86, 881–886. 10.1644/1545-1542(2005)86[881:EHACPR]2.0.CO;2

[B15] Schaller G. B. (2000). Wildlife of the Tibetan steppe. University of Chicago Press.

[B16] Seppey M. Manni M. Zdobnov E. M. (2019). BUSCO: assessing genome assembly and annotation completeness. Gene Predict. methods Protoc. 1962, 227–245. 10.1007/978-1-4939-9173-0_14 31020564

[B17] Signore A. V. Storz J. F. (2020). Biochemical pedomorphosis and genetic assimilation in the hypoxia adaptation of Tibetan antelope. Sci. Adv. 6 (25), eabb5447. 10.1126/sciadv.abb5447 32596473 PMC7299627

[B29] Smits T. H. M. (2019). The importance of genome sequence quality to microbial comparative genomics. BMC Genomics 20 (1). 10.1186/s12864-019-6014-5 PMC6701015 31429698

[B30] Wang Z. Ma T. Ma J. Han J. Ding L. Qiu Q. (2015). Convergent evolution of SOCS4 between yak and Tibetan antelope in response to high-altitude stress. Gene 572 (2), 298–302. 10.1016/j.gene.2015.08.024 26275942

[B18] Xu S. Q. Yang Y. Z. Zhou J. Jin G. E. Chen Y. T. Wang J. (2005). A mitochondrial genome sequence of the Tibetan antelope (*Pantholops hodgsonii*). Genomics, Proteomics Bioinforma. 3 (1), 5–17. 10.1016/s1672-0229(05)03003-2 PMC5172476 16144518

[B19] Zhang F. Jiang Z. Xu A. Zeng Y. Li C. (2013). Recent geological events and intrinsic behavior influence the population genetic structure of the chiru and Tibetan gazelle on the Tibetan plateau. PLoS ONE 8 (4), e60712. 10.1371/journal.pone.0060712 23637761 PMC3634780

[B20] Zhao S. Shetty J. Hou L. Delcher A. Zhu B. Osoegawa K. (2004). Human, mouse, and rat genome large-scale rearrangements: stability versus speciation. Genome Res. 14 (10a), 1851–1860. 10.1101/gr.2663304 15364903 PMC524408

[B21] Zhao Z. Zhou Y. Wang S. Zhang X. Wang C. Li S. (2020). LDscaff: LD-based scaffolding of *de novo* genome assemblies. BMC Bioinforma. 21 (S21), 570. 10.1186/s12859-020-03895-7 PMC7768660 33371875

