# Supplementary figures and images for: Uncovering rearrangements in the Tibetan antelope via population-derived genome refinement and comparative analysis with homologous species
Source: Front Genet. 2024 Feb 15;15:1302554. doi: 10.3389/fgene.2024.1302554 (PMC10902437; doi:10.3389/fgene.2024.1302554)

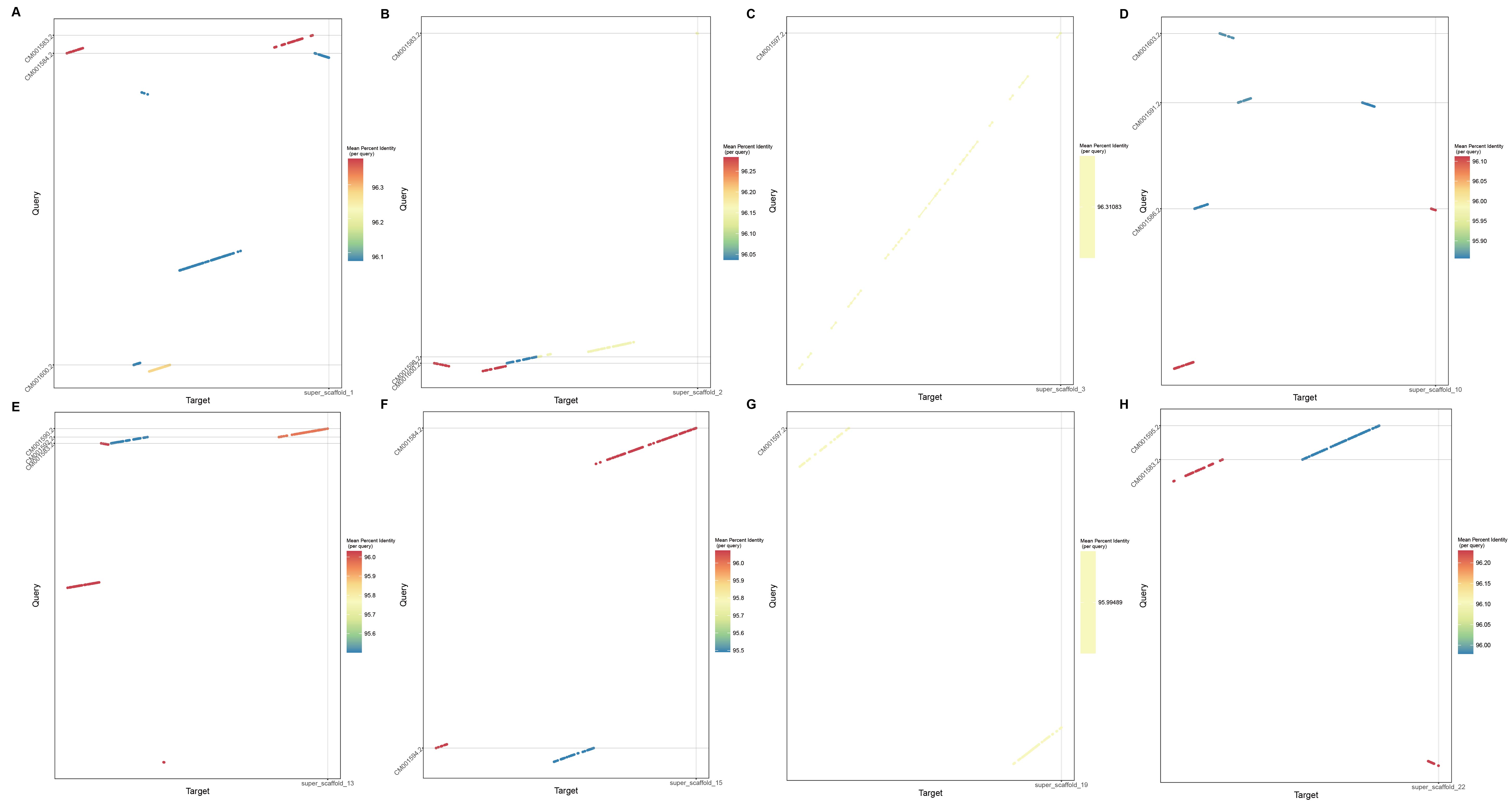

Supplement: Supplementary file 2 [file Image3.JPEG]

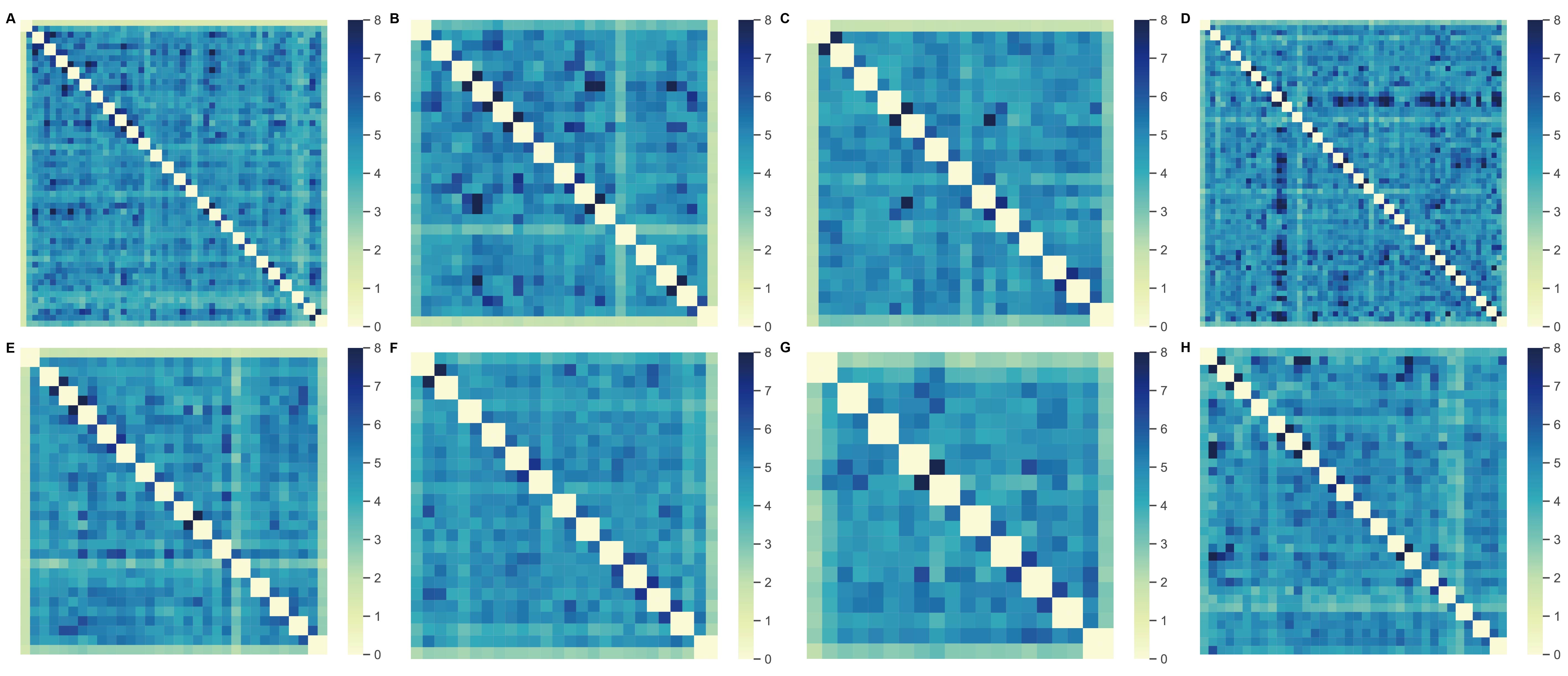

Supplement: Supplementary file 4 [file Image1.JPEG]

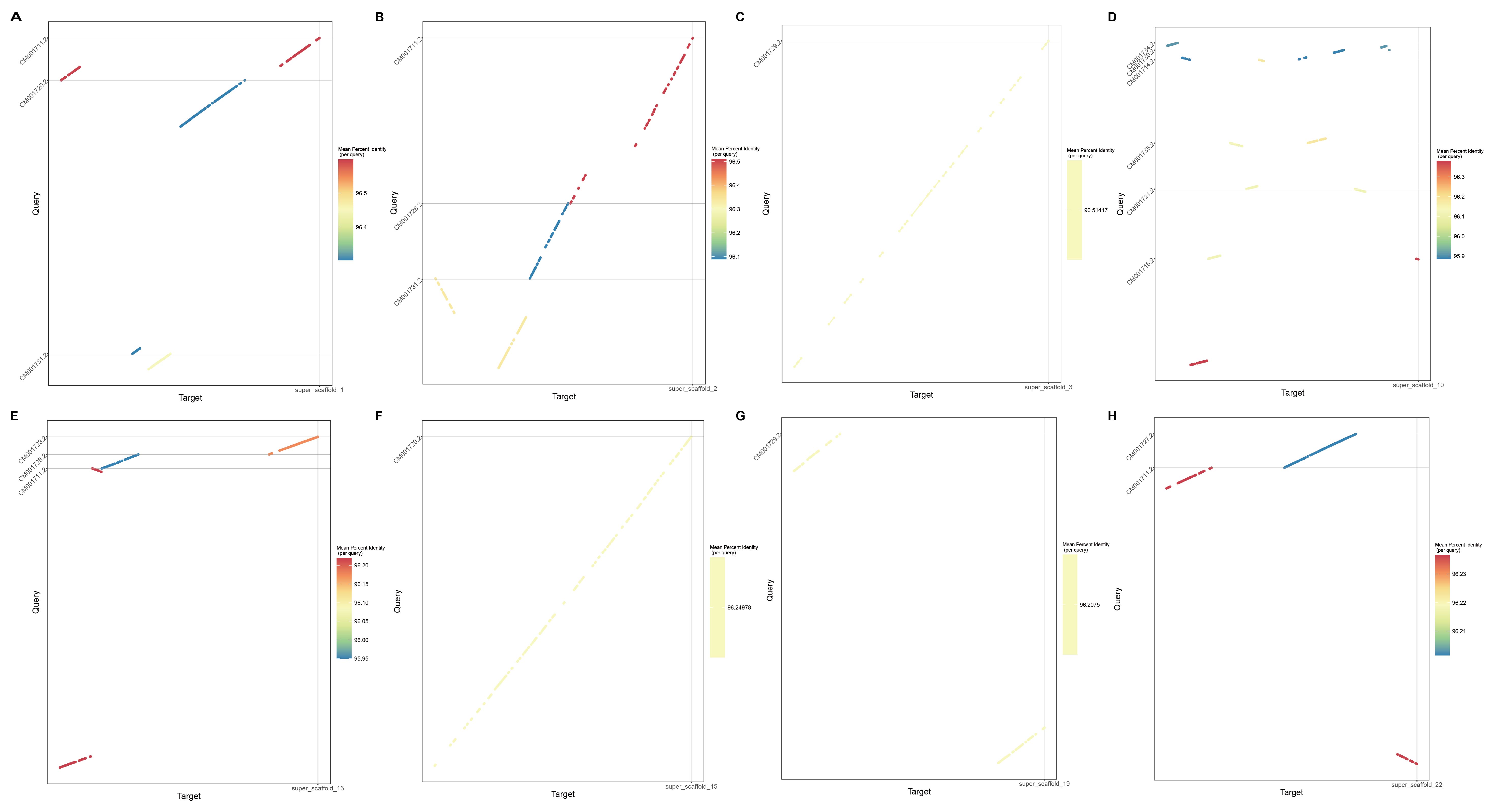

Supplement: Supplementary file 5 [file Image2.JPEG]
